# Limited Regeneration Potential with Minimal Epicardial Progenitor Conversions in the Neonatal Mouse Heart after Injury

**DOI:** 10.1016/j.celrep.2019.06.003

**Published:** 2019-07-02

**Authors:** Weibin Cai, Jing Tan, Jianyun Yan, Lu Zhang, Xiaoqiang Cai, Haiping Wang, Fang Liu, Maoqing Ye, Chen-Leng Cai

**Affiliations:** 1 Department of Developmental and Regenerative Biology, The Black Family Stem Cell Institute, Icahn School of Medicine at Mount Sinai, One Gustave L. Levy Place, New York, NY 10029, USA; 2 Department of Biochemistry, Guangdong Engineering & Technology Research Center for Disease-Model Animals, Zhongshan Medical School, Sun Yat-sen University, Guangzhou 510080, Guangdong Province, China; 3 Riley Heart Research Center, Herman B. Wells Center for Pediatric Research, Indiana University School of Medicine, 1044 West Walnut Street, Indianapolis, Indiana 46202, USA; 4 Cardiovascular Center, Children’s Hospital of Fudan University, Shanghai 201102, China; 5 Shanghai Key Laboratory of Clinical Geriatric Medicine, Department of Cardiology, Huadong Hospital Affiliated to Fudan University, Shanghai 200040, China; 6 These authors contributed equally; 7 Lead Contact

## Abstract

The regeneration capacity of neonatal mouse heart is controversial. In addition, whether epicardial cells provide a progenitor pool for *de novo* heart regeneration is incompletely defined. Following apical resection of the neonatal mouse heart, we observed limited regeneration potential. Fate-mapping of *Tbx18^MerCreMer^* mice revealed that newly formed coronary vessels and a limited number of cardiomyocytes were derived from the T-box transcription factor 18 (Tbx18) lineage. However, further lineage tracing with *SM-MHC^CreERT2^* and *Nfactc1^Cre^* mice revealed that the new smooth muscle and endothelial cells are in fact derivatives of pre-existing coronary vessels. Our data show that neonatal mouse heart can regenerate but that its potential is limited. Moreover, although epicardial cells are multipotent during embryogenesis, their contribution to heart repair through “stem” or “progenitor” cell conversion is minimal after birth. These observations suggest that early embryonic heart development and postnatal heart regeneration are distinct biological processes. Multipotency of epicardial cells is significantly decreased after birth.

## In Brief

The regeneration potential of the newborn mouse heart is controversial, and whether epicardial cells provide progenitors for coronary vascular regeneration is unclear. Cai et al. demonstrate a limited regeneration capacity of the neonatal heart upon injury. Epicardial cells do not convert into functional cardiac cells, including coronary vessels, during repair.

## Graphical Abstract

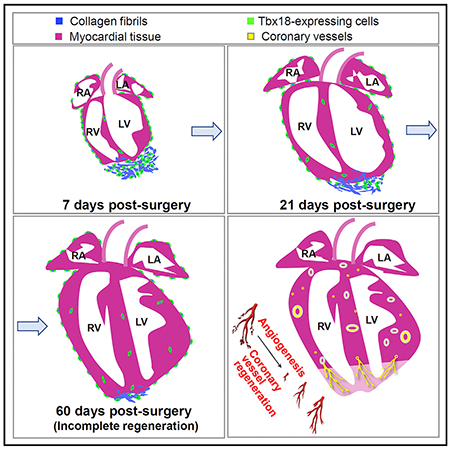

## INTRODUCTION

Acute myocardial infarction from coronary artery occlusion and ischemia is a major cause of death worldwide. Lack of regeneration with fibrogenic response to the acute injury is the main obstacle in treatment of cardiovascular disease (Laflamme and Murry, 2011). Replacing the scarred tissue with new functional cardiac cells is a potential therapeutic strategy to patients with heart failure (Jopling et al., 2010; Poss et al., 2002).

Although the mammalian heart lacks sufficient regeneration capacity at adulthood, a few studies have shown that the neonatal mouse heart can fully regenerate after injury (Bryant et al., 2015; Haubner et al., 2012; Porrello et al., 2011; Strungs et al., 2013). Similar to the teleosts, injury-induced neonatal mouse heart regeneration is initiated with rapid clotting and inflammatory and epicardial activation (Han et al., 2015; Lepilina et al., 2006; Porrello et al., 2011; Uygur and Lee, 2016). These observations revealed that within a short time window after birth, the mammalian hearts retain robust regeneration capacity (Haubner et al., 2012; Porrello et al., 2011). However, arguments arose recently concerning this capacity (Andersen et al., 2014; Andersen et al., 2016; Bryant et al., 2015; Kotlikoff et al., 2014; Polizzotti et al., 2016; Polizzotti et al., 2015), as they conversely reported that neonatal mouse hearts lack regeneration potential following apical resection or cryoinjury.

Despite the regeneration capacity of neonatal mouse heart, stimulation renewal of adult cardiac muscles remains challenging (Cai et al., 2003; Laflamme and Murry, 2011; Leferovich et al., 2001; Poss et al., 2002). Thus, identifying an optimal source of cardiac stem cells that can differentiate into different types of cardiac cells has been deemed an alternative approach for heart repair (Beltrami et al., 2003; Dawn et al., 2005; Orlic et al., 2001). Epicardial cells are multipotent cardiac progenitor cells during embryonic development (Cai et al., 2008; Limana et al., 2007; Zhou et al., 2008). In zebrafish and mice, a fetal epicardial gene expression program is immediately activated following cardiac injury (Han et al., 2015; Lepilina et al., 2006; Limana et al., 2010; Limana et al., 2007; Porrello et al., 2011; Uygur and Lee, 2016). However, it failed to identify epicardium-derived cardiomyocytes during heart regeneration in zebrafish (Kikuchi et al., 2011). In mice, it is not fully determined whether postnatal epicardial cells can differentiate into different cardiac lineages after injury. With Wilms tumor suppressor 1 (Wt1) lineage tracing of the adult epicardial cells, van Wijk et al. (2012) detected myocardial cells derived from epicardial mesenchyme post-infarction. Smart et al. (2011) also identified epicardial differentiation into cardiomyocytes, and this differentiation potential is enhanced upon thymosin β4 treatment. In contrast, Zhou et al. (2011) showed that epicardial cells only benefit heart repair through paracrine effects. Besides these unsolved critical issues regarding the epicardial myogenic potential, it is still uncertain whether postnatal epicardial cells sustain their potency and provide precursors for neovascularization during heart repair (Cai et al., 2008; Katz et al., 2012; Limana et al., 2007; Mikawa and Fischman, 1992; Mikawa and Gourdie, 1996; Zhou et al., 2008).

In this study, to define the regeneration potential and epicardial “progenitor” or “stem” activity in the injured neonatal mouse heart, we scrutinized the heart with apical resection. Through an inducible *Tbx18-MerCreMer* (*Tbx18*^*MerCreMer*/+^) knockin allele that labels epicardial cells, we observed that although the neonatal hearts can regrow, their regenerative potential is restricted. We further uncovered that smooth muscle cells within the newly formed coronary vessels are derived from transcription factor T-box 18 (Tbx18) and smooth muscle myosin heavy chain (SM-MHC) cells through angiogenesis. The new coronary endothelial cells are also derivatives of the pre-existing cardiac endothelium. While Tbx18 epicardial cells can give rise to cardiomyocytes, the number is exceedingly low and appears not to substantially contribute to a functional heart repair.

## RESULTS

### Extended Repair Process in the Neonatal Mouse Heart after Apex Amputation

To investigate neonatal mouse heart regeneration, we resected the lower portion of ventricular apex (~10% of the heart) at postnatal day 1 (P1) (Porrello et al., 2011). After amputation, the blood clotted immediately and the entire apex was sealed 3 hours after injury (Figure S1). Criteria to determine heart regeneration were based on several key factors including smoothness and thickness of apex, apical shape (curved or flat), and the presence of fibrotic tissues in the injured area.

**Figure 1.**
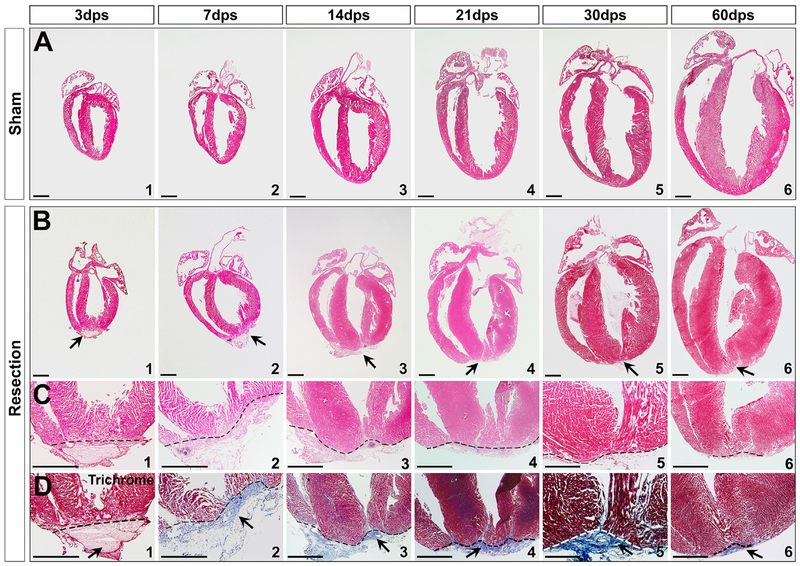
Neonatal Heart Repair upon Ventricular Apex Resection (A and B) Transverse sections of sham (A) and apex-resected hearts (B) at 3, 7, 14, 21, 30, and 60 dps. (C and D) High magnification of apex indicates amputation and clot at 3 dps (C1 and D1), and repair at 7–60 dps (C2–C6 and D2–D6). Trichrome staining of serial sections revealed that the apex was sealed with a large amount of fibrin between 7–30 dps (D2–D5), and fibrosis was replaced by the reconstituted wall by 60 dps (D6). Arrows indicate injury site. Scale bar, 1 mm. See also Figures S1 and S2.

**Figure 2.**
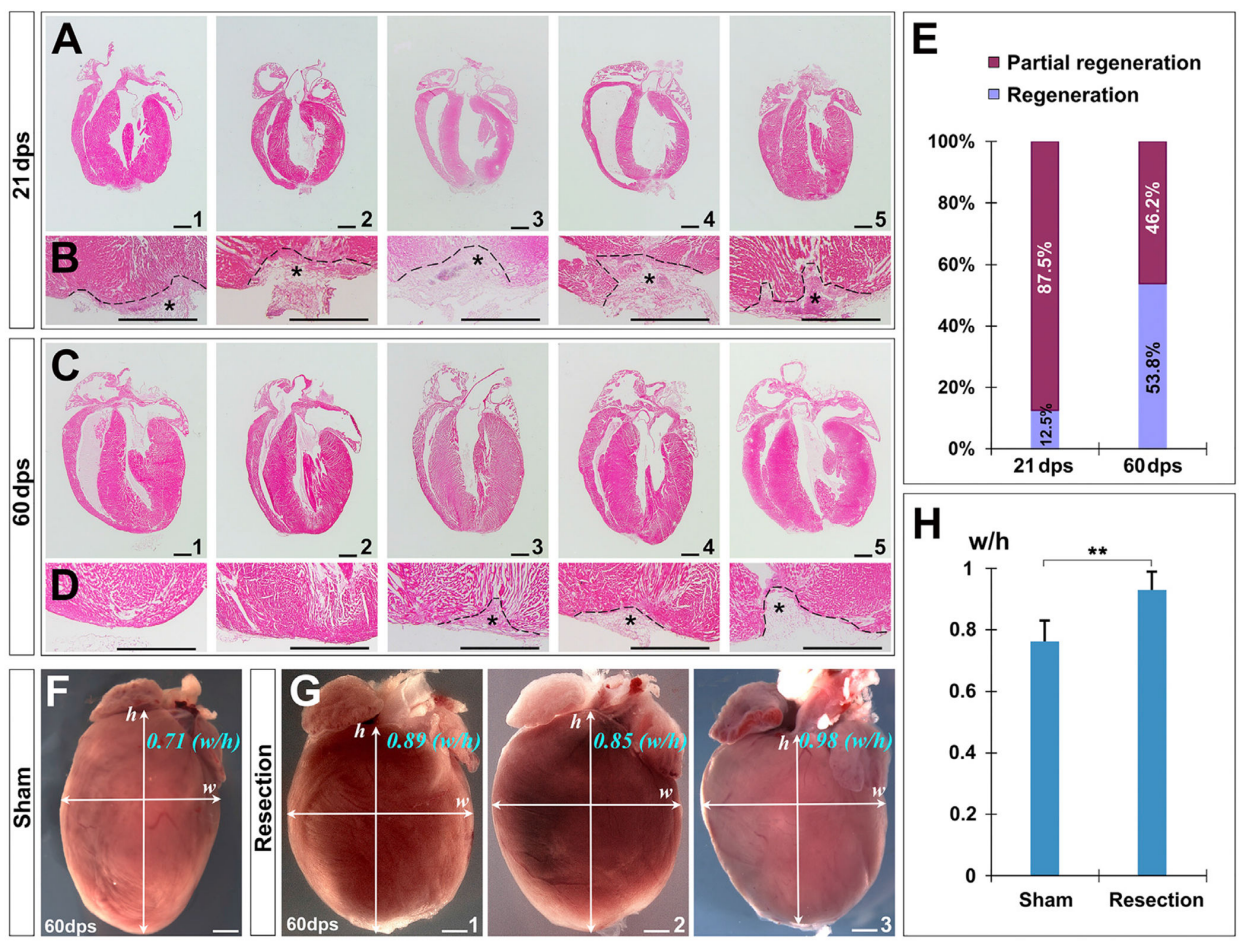
Limited Potential of Neonatal Heart Regeneration (A–D) Transverse sections of representative hearts at 21 dps (A and B) and 60 dps (C and D), suggesting that the repairing process takes longer than 21 days. A significant portion of hearts (46.2%) cannot be fully regenerated at 60 dps (C4 and C5). Asterisks indicate injured regions and incomplete repair. (E) Quantification of repair degree at 21 dps and 60 dps determined by apex shape and fibrotic tissues. (F–H) Global view of hearts in sham (F) and surgery (G) groups. Ventricular morphology is quantitated by the ratio of maximal horizontal width (w) to maximal vertical height (h) (H). **p < 0.01 versus the sham control. Scale bar, 1 mm. See also Figures S1 and S2.

We first examined the hearts at 7 days post-surgery (dps). Scars with rugged edges were observed (Figure S2). Haemotoxylin & Eosin (H&E) and trichrome staining were performed to determine how neonatal heart responds to amputation. Deposition of extracellular matrix was evident in the injured area at an early stage (Figure 1D2). After 7 dps, progressive repair with myocardial restoration was detected in the apex (Figure 1, 14–60 dps). However, by examining 59 hearts at 21 dps, we found that the time needed for repair was much longer than that which was previously reported (Porrello et al., 2011). Of 32 representative hearts, 28 (87.5%) still had substantial fibrotic tissues in the apex (Figure 1D4 and Figures 2A, B, and E), suggesting most hearts were not fully regenerated by 21 dps, in contrast to that which was suggested previously (Porrello et al., 2011).

We then examined the injured hearts at later stages. In 108 hearts collected at 60 dps, 26 were subjected to further histological analysis. Although 53.8% of them (14/26; Figure 2E) showed reconstruction with residual fibrotic tissues (Figure 1D6 and Figures 2C1, 2C2, 2D1, and 2D2; Figure S2B6), a large number (46.2%, 12/26; Figure 2E) still did not exhibit full regeneration. Damage to this group seems permanent because their apical shape did not retrieve (Figures 2C3–2C5 and 2D3–2D5; Figure S2B6). Substantial fibrotic tissues were persistently present in the injured region of these hearts when we examined at 90–180 dps and later (data not shown). Moreover, when we inspected the “regenerated” group hearts at 60 dps by comparing them with the sham group, it showed that although the apical edge of hearts in this group was relatively smooth with minimal fibrotic tissues, the global morphology was altered to a more “rounded” shape, characterized by significantly increased ventricular horizontal width to vertical height ratio (Figures 2F–2H).

### Severe Injury Promotes Myocardial Proliferation after 21 dps

**Figure 3.**
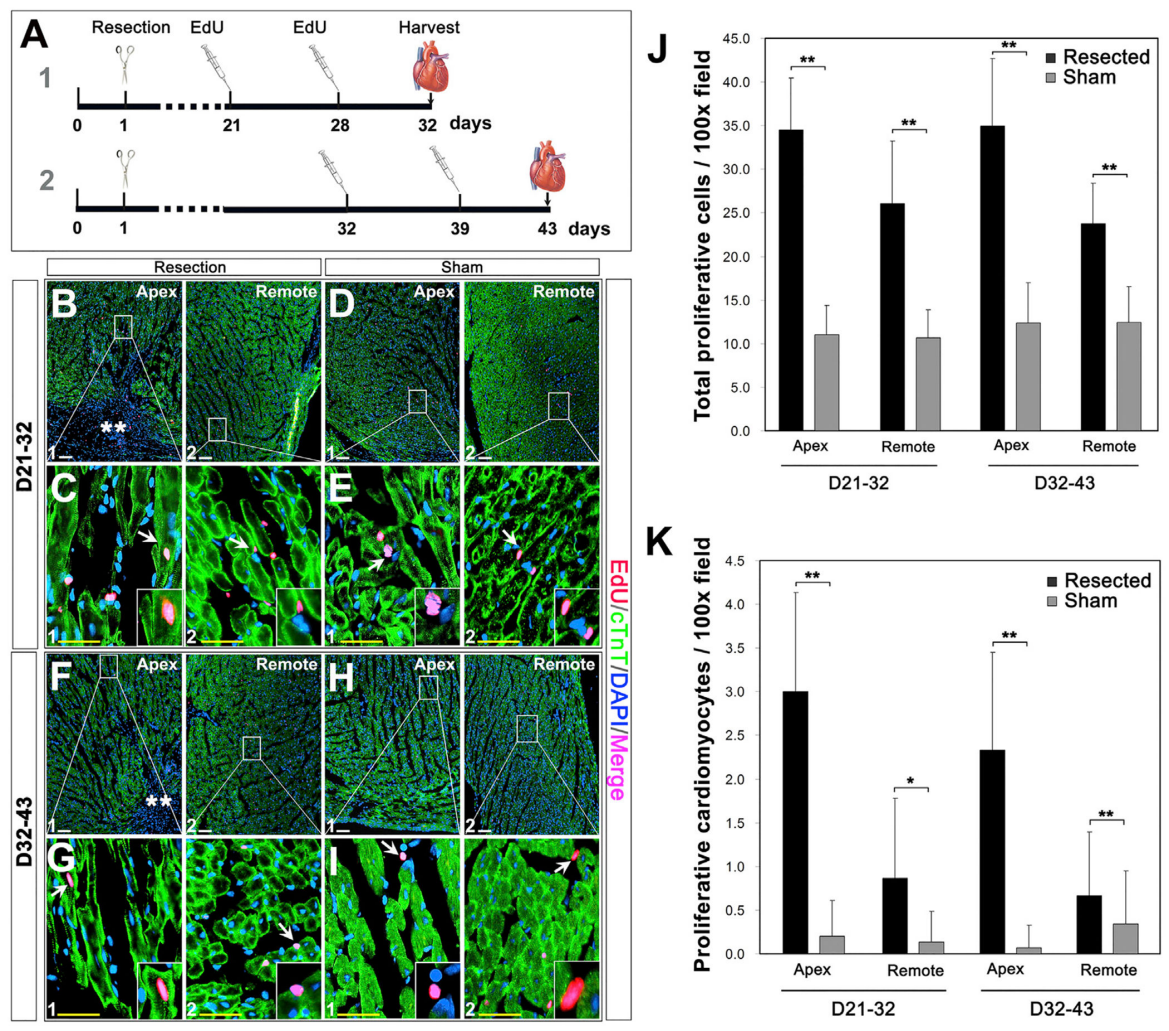
Cell Proliferation in the Injured Hearts (A) Diagram of pulse-chase EdU labeling of proliferating cells and the timeline for EdU injection and tissue collection. (B–I) Co-localization of EdU, cTNT, and nuclei (DAPI) in the apex (B1, D1, F1, and H1) and remote areas (B2, D2, F2, and H2) at 21–32 dps (B–E) and 32–43 dps (G–I) in the resected (B, C, F, and G) and sham (D, E, H, and I) groups. Asterisks indicate injury site. Arrows in (C1) and (G1) show EdU-positive cardiomyocytes, and arrows in (C2), (E), (G2), and (I) show EdU-positive non-cardiomyocytes (cTnT^−^). (J and K) Quantification of EdU-labeled proliferative cells (J) and cardiomyocytes (K) in sham and resected hearts at 32 dps and 43 dps. Quantitative analysis was performed on 5 fields at 100× magnification from 3 different hearts per group at each time point. *p<0.05, **p < 0.01 versus the control. Scale bar, 100 μm (white) and 25 μm (yellow). See also Figure S3.

Mammalian myocardial cells progressively decrease their proliferation activity after birth, with <1% turnover rate per year at adulthood (Bergmann et al., 2009; Li et al., 1996; Mollova et al., 2013; Naqvi et al., 2014; Senyo et al., 2013; Soonpaa and Field, 1997; Soonpaa et al., 1996; Walsh et al., 2010; Zhang and Kühn, 2014). In mice, cardiomyocytes maintain their cell cycle till 21 days after birth, and it showed that the neonatal heart fully regenerated with myocardial turnover by 21 dps (Porrello et al., 2011). We decided to assess the course of myocardial repair after 21 dps (Figures 2C–2E), given the improved repair rate or degree at 21–60 dps (Figure 2E). A 5-ethynyl-2’-deoxyuridine (EdU) pulse-chase assay was conducted to determine if myocardial proliferation continually contribute to heart repair after 21 dps. Mice were injected intraperitoneally with EdU at P21/P28 and P32/39, respectively (Figure 3A). Proliferative myocardial cells were identified by colocalization of EdU, cardiac troponin T (cTnT), and DAPI at 32 dps and 43 dps. Compared with the sham group, an increased number of EdU-positive (EdU^+^) cells were detected in the apex and remote region of injured hearts (32 dps: ~34.5 cells and ~26.1 cells/100× field in apex and remote region versus ~11.0 cells and ~10.7 cells/100× field in the sham apex and remote region; 43 dps: ~34.9 cells and ~23.7 cells/100× field in the injured apex and remote region versus ~12.1 cells and ~12.5 cells/100× field in the sham apex and remote region, Figures 3B–J), suggesting that the apical injury promotes global cardiac cell proliferation at 21–43 dps, although most of them are non-cardiomyocytes (cTnT^−^; Figures 3C2 and 3G2). Further evaluation revealed an increased number of EdU^+^ cardiomyocytes in the injured apex border zone at 32 dps and 43 dps (~3.0 cells and ~2.3 cells/100× field, respectively) (Figures 3C1, 3G1, and 3K), and the number was significantly higher than that of the sham-operated controls (~0.2 cells and ~0.07 cells/100× field at 32 dps and 43 dps, respectively) (Figures 3E1, 3I1, and 3K). The plasma membrane marker wheat germ agglutinin (WGA) was used to identify the true proliferation of cardiomyocytes as opposed to binucleation (Figure S3). These observations indicate that severe injury can induce myocardial proliferation after 21 dps.

### Tbx18-Expressing Cells Contribute to Heart Repair

**Figure 4.**
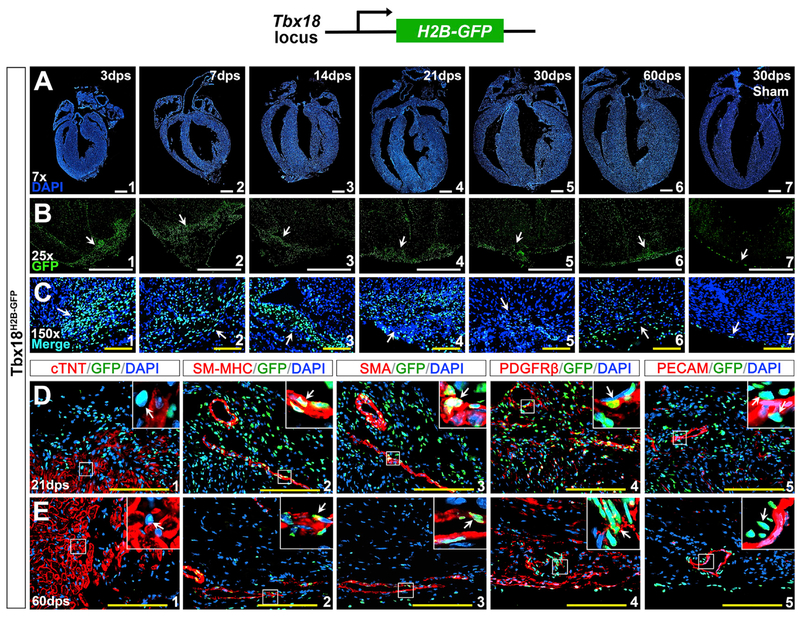
Accumulated Tbx18-Expressing Cells in the Injured Region (A) DAPI staining at 3, 7, 14, 21, 30, and 60 dps. (B and C) Robust Tbx18-expressing cells (*Tbx18^H2B-GFP^*-positive) in the injury site during repair (arrows) with high density at 3–21 dps (B1–B4 and C1–C4). (D and E) Immunostaining in the injured site at 21 dps (D) and 60 dps (E). *Tbx18^H2B-GFP^* is not co-expressed with cTNT (D1 and E1) but is co-expressed with SM-MHC (D2 and E2), α-SMA (D3 and E3), and PDGFRβ (D4 and E4). *Tbx18^H2B-GFP^* is not co-expressed with PECAM in the endothelial cells (D5 and E5). The top right corner images in (D) and (E) are high magnification of the areas outlined in each panel. Scale bar, 1mm (white) and 100 μm (yellow). See also Figures S4 and S5.

The transcription factor Tbx18 is expressed in the epicardium across species in vertebrates (Haenig and Kispert, 2004; Kraus et al., 2001). Previous studies suggested that epicardial cells are cardiac progenitors and give rise to multiple cardiac lineages, including coronary smooth muscle cells (cSMCs), endothelium, fibroblasts, and cardiomyocytes, during embryonic heart formation (Cai et al., 2008; Katz et al., 2012; Mikawa and Fischman, 1992; Mikawa and Gourdie, 1996; Zhou et al., 2008). To determine the potential *de novo* contribution of epicardial progenitor cells through differentiation in heart repair, we performed apical resection on *Tbx18*^*H2B-GFP*/+^ mice at P1. H2B-GFP in *Tbx18*^*H2B-GFP*/+^ knockin reporter mice recapitulates endogenous Tbx18 expression (Cai et al., 2008), and the mice carrying heterozygous null for *Tbx18* are normal in heart formation and function (Wu et al., 2013). Upon surgical amputation, massive *Tbx18^H2B-GFP^*-positive cells accumulated in the ventricular apex at 3 dps (Figures 4A1, 4B1, and 4C1). Density of these GFP^+^ cells was high at 1–21 dps (Figures 4A1–4A4, 4B1–4B4, and 4C1–4C4) and slowly decreased after 30 dps (Figures 4A5, 4A6, 4B5, 4B6, 4C5, and 4C6). To determine the cause of reduction of Tbx18^+^ cells during heart repair, we performed TUNEL (terminal deoxynucleotidyl transferase dUTP nick-end labeling) staining on the injured heart. Apoptotic Tbx18^+^ cells were detected in the injured area throughout the heart repair process (Figure S4), indicating apoptosis could be the cause of reduction. An increased number of Tbx18^+^ cells were not detected in the sham group (Figures 4A7, 4B7, and 4C7).

We further performed immunostaining to define cell type of these Tbx18^+^ cells. With tissues at all stages tested (3, 7, 14, 21, 30, and 60 dps), *Tbx18^H2B-GFP^*-positive cells were not co-expressed with cTnT or platelet endothelial cell adhesion molecule (PECAM/CD31), indicating they are not cardiomyocytes or endothelial cells (Figures 4D1, 4D5, 4E1, and 4E5). In contrast, a subpopulation of *Tbx18^H2B-GFP^*-positive cells were co-expressed with smoothel muscle α-actin (α-SMA), SM-MHC, or platelet-derived growth factor receptor beta (PDGFRβ), which are markers for vascular smooth muscle cells (SMCs) and pericytes (Figures 4D2–4D4 and 4E2–4E4). We also found that some Tbx18^+^ cells were co-expressed with CD90 and PDGFRα, markers for cardiac fibroblast (Acharya et al., 2012; Hudon-David et al., 2007; Ivey and Tallquist, 2016; Smith et al., 2011; Travers et al., 2016) (Figure S5). This demonstrates that at least a subpopulation of Tbx18-expressing cells are SMCs and pericytes and a portion of Tbx18^+^ cells are fibroblasts in the injured area.

**Figure 5.**
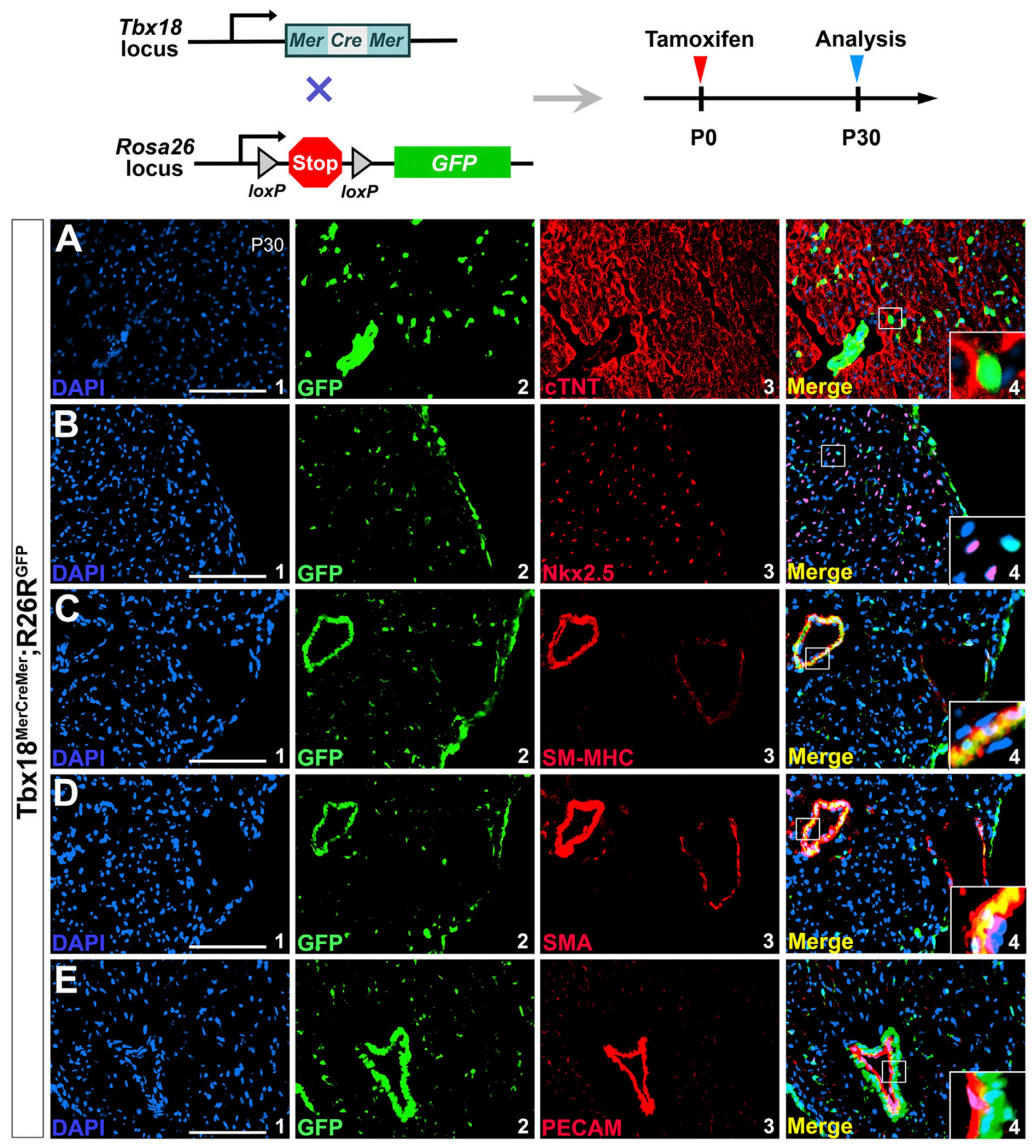
Tbx18^+^ Cells Do Not Become Cardiomyocytes or Coronary Endothelial Cells after Birth The neonatal mice were given a single subcutaneous dose of tamoxifen at P0, and the hearts were collected at postnatal day 30 for analysis. (A and B) Tbx18 progeny do not include cardiomyocytes (not co-localized with cTNT, A, or Nkx2.5, B). (C and D) Co-expression of Tbx18 progeny with SM-MHC (C4) and α-SMA (D4). (E) Tbx18 lineage is not co-expressed with PECAM in the coronary endothelial cells (E4). (A1), (B1), (C1), (D1), and (E1) are DAPI staining. GFP-positive cells in (A2), (B2), (C2), (D2), and (E2) are cells of Tbx18 lineage. (A3), (B3), (C3), (D3), and (E3) are antibody staining for cTNT, Nkx2.5, SM-MHC, α-SMA and PECAM, respectively. (A4), (B4), (C4), (D4), and (E4) are overlays of (A1–A3), (B1–B3), (C1–C3), (D1–D3), and (E1–E3), respectively. Scale bar, 100 μm. See also Figure S6.

We next attempted to determine the differentiation potential of Tbx18^+^ cells to heart repair with a *Tbx18*^*MerCreMer*/+^ knockin allele, in which a *MerCreMer* cassette was inserted into the start codon of Tbx18 to induce Cre expression under full *Tbx18* regulatory elements upon tamoxifen induction (Figure S6A). To avoid interference by cardiomyocytes from embryonic epicardial cells during heart development (Cai et al., 2008; Zhou et al., 2008), *Tbx18*^*MerCreMer*/+^ mice were crossed with *R26R^GFP^* reporter mice and the compound heterozygous (*Tbx18*^*MerCreMer*/+^;*R26R^GFP^*) mice were injected with tamoxifen at P0 (Figure 5). This enabled us to specifically label Tbx18-expressing cells after birth. Cre efficiency was confirmed by robust GFP expression in the limb of the newborn *Tbx18*^*MerCreMer*/+^;*R26R^GFP^* mice after tamoxifen administration (Figure S6C). With cardiac tissues collected at P30, we did not detect co-localization of GFP with myocardial markers cTnT and Nkx2.5 or endothelial cell marker PECAM (Figure 5). However, co-expression of GFP with SM-MHC and α-SMA was found. This further confirmed that Tbx18 is actively expressed in SMCs but not in endothelial cells or cardiomyocytes after birth, as illustrated with *Tbx18*^*H2B-GFP*/+^ reporter mice (Figure 4).

**Figure 6.**
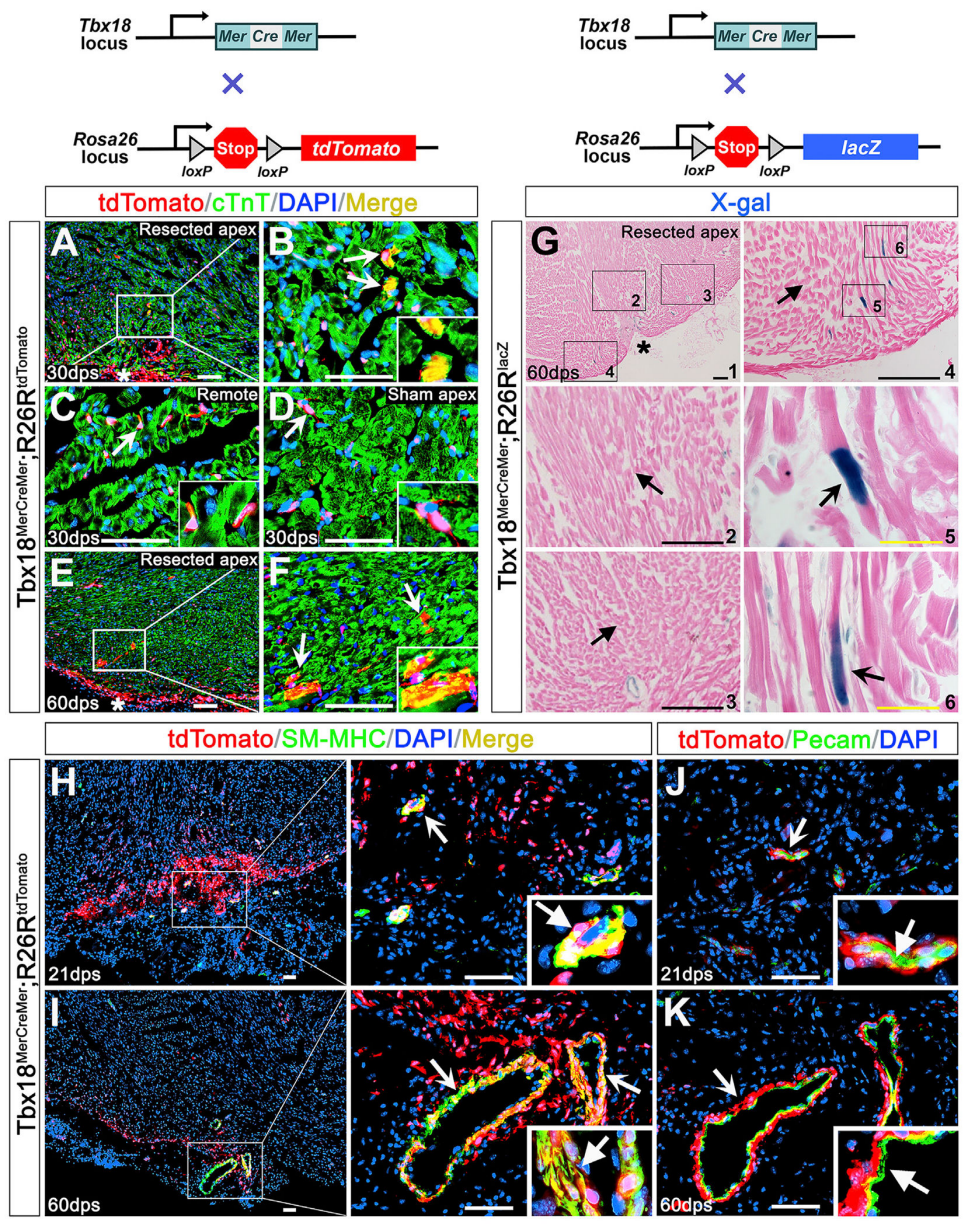
Tbx18 Lineage Analysis after Cardiac Injury (A–K) *Tbx18^MerCreMer^*;*R26R^tdTomato^* (A–F and H–) and *Tbx18^MerCreMer^*;*R26R^lacZ^* (G) mice were injected with tamoxifen after apex resection. (A–F) Immunostaining was performed on *Tbx18^MerCreMer^*;*R26R^tdTomato^* hearts at 30 dps (A–D) and 60 dps (E and F). Arrows in (A), (B), (E), and (F) indicate tdTomato and cTNT double-positive cells in the apex at 30 dps (A and B) and 60 dps (E and F). Arrows in (C) and (D) indicate non-myocardial Tbx18 lineage (cTnT^−^) in the remote area (C) or in the apex of sham group (D). (B–D and F) Right-bottom corner images are high magnification of the areas indicated by arrows. (G) X-gal staining of *Tbx18^MerCreMer^*;*R26R^lacZ^* hearts revealed Tbx18-derived cardiomyocytes (arrows in G4–G6) in the apex at 60 dps. (G2), (G3), and (G4) are high magnification of the square areas in G1. (G5) and (G6) are high magnification of the square areas in (G4). Unnotched arrows (G2–G4) indicate X-gal negative cells. (H–K) Immunostaining on *Tbx18^MerCreMer^*;*R26R^tdTomato^* hearts at 21 dps (H) and 60 dps (I). tdTomato signals (arrows) are co-localized with SM-MHC in the apex at 21 dps and 60 dps (H and I). PECAM staining (unnotched arrows in J and K) is not co-localized with tdTomato (arrows) in the regenerative coronary at 21 dps and 60 dps. Right bottom corner images (H-K) are high magnification of areas indicated by notched arrows. Scale bar, 50 μm (white), 100 μm (black) and 20 μm (yellow). See also Figure S6.

To trace the progeny of Tbx18-expressing cells after injury, *Tbx18*^*MerCreMer*/+^;*R26R*^*tdTomato*/+^ compound heterozygous mice were subjected to apex resection followed by tamoxifen administration (Figure 6). With the hearts harvested at 30 dps and 60 dps, we did not detect any Tbx18-derived cardiomyocytes (cTnT^+^/tdTomato^+^ double positive) in areas distant from the injured area (Figure 6C) or in the sham group hearts (Figure 6D). A few cTnT^+^ cardiomyocytes were found in the injured apex at both stages examined (Figures 6A, 6B, 6E, and 6F). However, the number is extremely low (fewer than 30 cells in the entire injured region). To verify this, a parallel study with *Tbx18*^*MerCreMer*/+^;*R26R*^*lacZ*/+^ mice was performed. Cardiomyocytes from Tbx18 lineage (lacZ^+^) were detected in the newly formed apex at 60 dps (Figures 6G4–6G6) and the incidence was also remarkably rare (fewer than 35 cells in the whole heart). This revealed that although Tbx18^+^ cells can turn into cardiomyocytes, their potential is exceedingly low in the injured heart. Most cardiomyocytes are not progeny of Tbx18-expressing cells (Figures 6G2–6G4).

We further inquired if the newly formed cSMCs and endothelium are Tbx18 epicardial derivatives, given that epicardial progenitors give rise to cSMCs (Cai et al., 2008; Katz et al., 2012; Mikawa and Fischman, 1992; Mikawa and Gourdie, 1996; Zhou et al., 2008) and endothelium (Katz et al., 2012; Mikawa and Fischman, 1992; Zhou et al., 2008) during embryonic heart development. Surgeries, genetic tracing of Tbx18 lineage, and tamoxifen induction were performed on *Tbx18*^*MerCreMer*/+^;*R26R*^*tdTomato*/+^ mice. Upon immunohistochemistry with cardiac tissues collected at 21 dps and 60 dps, we detected co-localization of cSMCs (SM-MHC^+^) with tdTomato (Tbx18-expressing or progeny) in the apex (Figures 6H and 6I). In contrast, no co-localization of the PECAM^+^ coronary endothelium with tdTomato was found (Figures 6J and 6K), suggesting that cSMCs, but not endothelial cells, in the newly formed coronary vessels are derivatives of Tbx18-expressing cells.

### Angiogenic Process Contributes to the New Coronary Vessel Formation

The newly formed cSMCs could differentiate *de novo* from Tbx18^+^ epicardial cells or they could be derived from the pre-existing Tbx18^+^ cSMCs through angiogenesis, given that Tbx18 is actively expressed in both epicedial cells and cSMCs after birth (Figures 4 and 5). We therefore attempted to determine the ultimate origin of cSMCs in the new coronary vessels of injured hearts.

**Figure 7.**
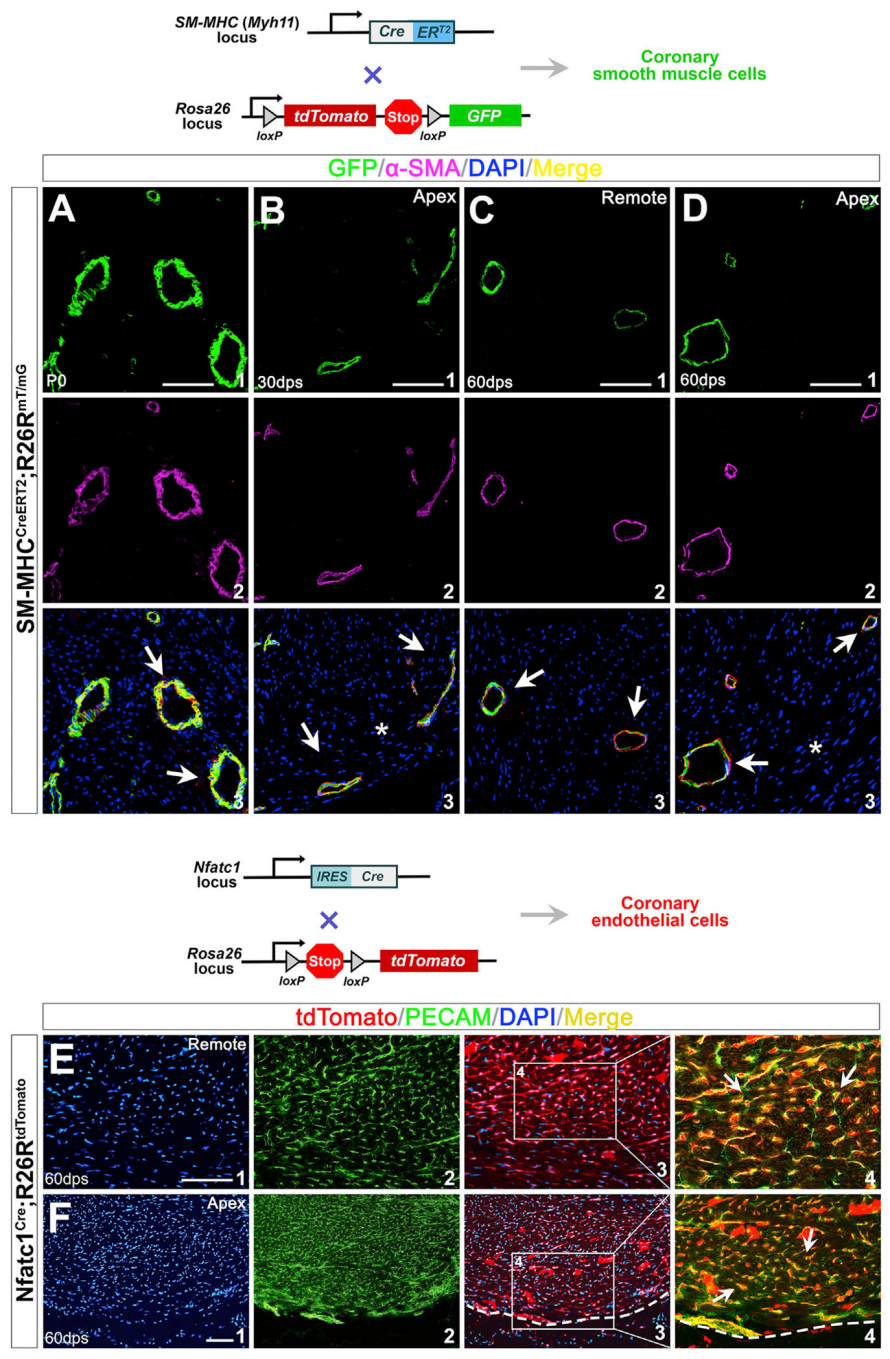
New Coronary SMCs and Endothelial Cells within the Ventricular Apex Are Derived from the Pre-existing Coronary Vessels (A) Immunostaining of *SM-MHC^CreERT2^*; *R26R^mT/mG^* hearts at P0 with tamoxifen induction during gestation. (B–D) Hearts at 30 dps (B) and 60 dps (C and D) with apical resection at P1. GFP^+^ cells (A1, B1, C1, and D1) are cells of SM-MHC lineage. (A2), (B2), (C2), and (D2) are antibody staining for α-SMA. (A3), (B3), (C3), and (D3) are overlays of SM-MHC lineage (GFP^+^) with DAPI and α-SMA. α-SMA^+^ SMCs in the injured apex are from SM-MHC lineage (arrows in B3 and D3). Asterisk indicates injured area. (E and F) Regeneration of coronary endothelial after apical resection. Immunostaining of *Nfatc1*^*Cre*/+^;*R26R*^*tdTomato*/+^ hearts at 60 dps. (E) Nfatc1 progeny give rise to coronary endothelium (arrows in E4). (F) PECAM^+^ endothelial cells in the newly formed apex are from Nfatc1^+^ endocardial/endothelial lineage (arrows in F4). (E1) and (F1) are DAPI staining; (E2) and (F2) are PECAM staining; (E3) and (F3) are overlays of Nfatc1 lineage (tdTomato^+^) with DAPI. (E4) and (F4) are high-magnification images of the areas outlined in (E3) and (F3). They are overlays of PECAM staining and Nfatc1 lineage (tdTomato^+^). Scale bar, 100 μm.

*SM-MHC-CreER^T2^* transgenic mice specifically label SMCs upon tamoxifen induction (Wirth et al., 2008). We crossed *SM-MHC-CreER^T2^* with *R26R^mT/mG^* (*R26R^tdTomato/GFP^*) mice and compound heterozygous animals (*SM-MHC-CreER^T2^*; *R26R^mT/mG^*) were injected with tamoxifen at E13.5 and E16.5. We found *SM-MHC-CreER^T2^* effectively labels cSMCs at birth (Figure 7A). Littermates were subjected to cardiac apical injury at P1. By immunohistochemistry of hearts collected at 30 dps and 60 dps, we detected that all the cSMCs (α-SMA^+^) in the remote area are GFP-positive (GFP^+^), indicating effective labeling of the pre-existing cSMCs. When examining the injured area, we observed that new SMCs are constantly GFP^+^ (Figure 7B–7D), demonstrating that they are derivatives of the pre-existing GFP^+^ cSMCs rather than through cell fate conversion from Tbx18^+^ epicardial cells.

As discussed above, Tbx18-expressing cells do not convert to coronary endothelium and the origin of a new coronary endothelium is inconclusive with *Tbx18*^*MerCreMer*/+^;*R26R*^*tdTomato*/+^ mouse models (Figures 6J and 6K). We then utilized *Nfatc1^Cre^* mice to mark pre-existing coronary endothelium. Nfatc1 (nuclear factor of activated T cells, cytoplasmic 1) is a transcription factor specifically expressed in the early endocardial precursors and sinus venosus before they become endothelium during embryogenesis (Chen et al., 2014; Tian et al., 2015; Wu et al., 2012). Nfatc1 is not expressed in the differentiated cardiac endothelium after birth (Chen et al., 2014; Tian et al., 2015; Wu et al., 2012). *Nfatc1^Cre^* progeny encompass most coronary endothelium during heart formation (Cai et al., 2013; Chen et al., 2014; Wu et al., 2012). By examining *Nfatc1*^*Cre*/+^;*R26R*^*tdTomato*/+^ hearts at P0, P30, and P60, we found Nfatc1 lineages constitute most PECAM^+^ coronary endothelium in the ventricular apex (Figure 7E; data not shown). We applied *Nfatc1*^*Cre*/+^;*R26R*^*tdTomato*/+^ mice to label the existing coronary endothelium, and surgical resection was performed on these animals at P1. With these animals, if tdTomato^−^ and PECAM^+^ coronary endothelial cells were present in the apex, it would indicate that they are not from pre-existing endothelial cells. In fact, we found PECAM^+^ cells in the injured area (including the border zone) were always tdTomato^+^ (60 dps, Figure 7F), suggesting that the pre-existing endothelial cells made complete contributions to the new coronary endothelial formation through angiogenesis (instead of through vasculogenesis from cardiac “progenitor” cells).

## DISCUSSION

### Regeneration Capacity of the Neonatal Mouse Heart Is Restricted

The regeneration potential of the neonatal mouse heart is controversial (Andersen et al., 2014; Andersen et al., 2016; Haubner et al., 2012; Kotlikoff et al., 2014; Polizzotti et al., 2015; Porrello et al., 2011; Sadek et al., 2014; Strungs et al., 2013). Previous report showed that, with surgical resection of 15% of ventricular myocardium in apex, the mouse heart can fully regenerate within 21 days (Porrello et al., 2011). Here, we show that most injured hearts (87.5%) had substantial fibrotic tissues at 21 days, demonstrating that the time needed for restoration is much longer than that reported (Porrello et al., 2011). Likewise, regeneration of resected zebrafish heart is not accomplished until after 60 days (Poss et al., 2002).

At 60 dps, we found only half of the injured hearts (53.8%) restored or regenerated with collagen residues, and a large portion (46.2%) still displayed incomplete repair with fibrotic tissues. Even in the “regenerated” group, overall morphology of the heart is inevitably changed to a “rounded” shape (Figure 2). A long-term follow up of cardiac regeneration at 180 dps showed that apex resection of P1 mice resulted in irreversible fibrosis and dilated cardiomyopathy (Andersen et al., 2014; Andersen et al., 2016). Our data support the notion that the newborn mouse heart can repair; however, its regeneration potential is apparently restricted but not unlimited (even with 10% ventricular apex amputation as demonstrated in this study). The prolonged repair process and variable regeneration capacity may be due to severity of the injury and actual surgical procedure to the hearts (Andersen et al., 2014; Bryant et al., 2015; Darehzereshki et al., 2015; Konfino et al., 2015). However, if the injury of this study is “more severe” than that which has been reported elsewhere, it should indicate that larger injuries do not necessarily result in a proportionally greater regenerative response but enhanced fibrosis (Bryant et al., 2015), further demonstrating “limited” regeneration capacity in the neonatal mouse heart. Nevertheless, it may be important to assess the regeneration potential of the neonatal mouse heart following small, large, superficial, and transmural infarcts in order to determine the physiological limits (Porrello and Olson, 2014). Moreover, with the increased restoration rate of the injured heart from 21 dps (12.5%) to 60 dps (53.8%), it appears that severe injury leads to an extended repairing time window (prolonged myocardial proliferation after 21 dps).

### Multipotency of Epicardial Cells Is Eliminated in Postnatal Heart Repair

Epicardial activation was found in zebrafish and mouse hearts upon acute injury (Huang et al., 2012; Kikuchi et al., 2010; Lepilina et al., 2006; Limana et al., 2010; Zhou et al., 2011). Our study consistently showed accumulation of Tbx18^+^ cells in the injured area upon apex resection. The multidirectional differentiation potential of epicardial cells during embryonic development (Cai et al., 2008; Kikuchi et al., 2011; Limana et al., 2007; Zhou et al., 2008) suggests they may act as progenitor cells in the neonatal and adult heart repair. As of today, whether postnatal epicardial cells are multipotent and contribute to heart repair is not fully defined. Lineage tracing with *Wt1^Cre^* mouse showed that Wt1^+^ epicardial cells differentiated into cardiomyocyte after myocardial infarction (van Wijk et al., 2012). Studies with the *Wt1-CreER^T2^* mouse model did not find conversion of cardiomyocytes from epicardial cells in the adult heart (Zhou et al., 2011). In this study, only a very limited number of cardiomyocytes from Tbx18^+^ cells were detected, suggesting that myocardial potential of these cells is extremely low and does not contribute to a functional myocardial repair after birth.

Epicardial cells are precursors that give rise to coronary SMCs and possibly endothelial cells during embryonic heart formation (Cai et al., 2008; Katz et al., 2012; Mikawa and Fischman, 1992; Mikawa and Gourdie, 1996; Zhou et al., 2008). Despite their potential during development, our lineage tracing results show that postnatal epicardial cells minimally convert to cardiac endothelium. *Nfatc1*^*Cre*/+^;*R26R*^*tdTomato*/+^ and *SM-MHC-CreER^T2^*;*R26R^tdTomato/GFP^* models suggest that new coronary vasculatures are derived from the pre-existing coronary endothelium and cSMCs after injury. These observations reveal that coronary formation during embryonic development and neovascularization upon injury are distinct biological processes. The multipotent nature of embryonic epicardial cells significantly decreases after birth.

In summary, our studies suggest a limited regeneration potential of the neonatal heart upon injury, and postnatal epicardial cells generally do not convert into functional cardiac cells during heart repair, including neovascularization. In the future, it will be of interest to compare embryonic, neonatal, and adult epicardial cells in order to define molecular basis underlying the change of their potency.

## STAR★METHODS

### LEAD CONTACT AND MATERIALS AVAILABILITY

Further information and requests for resources and reagents should be directed to and will be fulfilled by the Lead Contact, Chen-Leng Cai (chenleng@iu.edu).

### EXPERIMENTAL MODEL AND SUBJECT DETAILS

#### Animals

##### Wild-type CD1 mice were obtained from Charles River Laboratories.

Tbx18^H2B-GFP/+^, *SM-MHC^CreERT2^* and *Nfatc1^Cre^* mice were described previously (Cai et al., 2008; Wirth et al., 2008; Wu et al., 2012). To create *Tbx18*^*MerCreMer*/+^ knockin line, a *MerCreMer* cassette followed by a *Neomycin* resistance gene was introduced into the mouse *Tbx18* start codon through gene targeting. Animals derived from the positive embryonic stem cells were crossed to a *Flippase* deleter (*FLPe*) line to remove the *Neomycin* cassette (Rodríguez et al., 2000). Rosa26 reporter lines, including *R26R*^*lacZ*/+^,*R26R*^*GFP*/+^,*R26R*^*tdTomato*/+^ and *R26R^tdTomato/GFP^* (*R26R^mT/mG^* or *R26R^m-tdTomato/m-GFP^*) were obtained from Jackson Laboratories (Bar Harbor, ME) (Madisen et al., 2010; Muzumdar et al., 2007; Soriano, 1999). Genetically modified mouse models were bred in a Black Swiss background. Experiments with animal models were performed in accordance with the guidelines and approval of the Institutional Animal Care and Use Committee (IACUC) at the Icahn School of Medicine at Mount Sinai and Sun Yat-sen University Zhongshan Medical School.

##### Animal models with cardiac surgery

The apical resection of neonatal mouse hearts was performed as described by Porrello et al. with modifications (Porrello et al., 2011). Briefly, postnatal day 1 (P1) mice were anesthetized by cooling on an ice bed for 4 minutes, and subsequently a left lateral thoracotomy was made in the fourth or fifth intercostal space following skin incision. Under direct stereo-microscopic visualization, the pericardial sac was opened, and the heart apex was held with a 200 μL pipette tip connected to an adjusted bench vacuum. The ventricular apex was resected with iridectomy scissors when the left ventricular chamber was exposed. Approximately 10% (10.5 ± 1.81% of the total heart by weight) cardiac tissues were removed. After the injury, the animals were gently moved back to the ice bed to minimize bleeding, and then the thoracic wall incisions and the skin wound were sutured (7-0 silk suture). For sham group, a similar thoracotomy procedure was performed without apical resection. The postsurgical neonates were transferred onto a temperature-controlled pad (37°C) for several minutes. After recovery from anesthesia, the pups were put back with their mothers, and the wound generally healed in 4-5 days. Cardiac regeneration was evaluated with several criteria, including defects in the injury site, smoothness of apex edge, amount of fibrosis and thickness of the apex.

### METHOD DETAILS

#### Tamoxifen administration

Tamoxifen (T5648, Sigma-Aldrich, St Louis, MO, USA) was dissolved in ethanol (10%) and sesame oil (90%) solution to a final concentration of 10 mg/ml. Cre recombinase translocation was triggered by intraperitoneal injection of 30 μL tamoxifen solution in the neonatal pups (P0). For *SM-MHC-Cre^ERT2^*;*R26R^mT/mG^* reporter mouse, pregnant female mice were given tamoxifen by intraperitoneal injection with the dose of 100 μg/g body weight once when embryos were at E13.5 and E16.5. All newborn mice at P1 underwent apical resection.

#### Cardiac morphology analysis

For histology, dissected hearts were immersed in 10% potassium chloride for 5 min and allowed to stop at diastole. After washed in PBS, the hearts were fixed in 4% paraformaldehyde/PBS for 1-2 hours (10min fixation for the hearts with fluorescence proteins) and were then transferred into 20% sucrose/PBS at 4°C for 2 hours. Subsequently, hearts were embedded in TissueTek OCT compound. Cryosections were cut at 6-8 μm thickness using a Leica cryostat. Hematoxylin/eosin staining was performed with standard procedures (Cai et al., 2013). Masson’s trichrome staining was performed with a kit from Sigma (HT15-1KT).

For global cardiac morphology and shape assessment, the hearts were immediately immersed in 15% potassium chloride solution after dissection. Ventricular maximal horizontal diameter (width, w) and vertical diameter (height, h) was measured on the whole-mount images in Photoshop CS software (Adobe, CA, USA). Ratio (w/h) is calculated for quantitative and statistical analysis.

#### Immunohistochemistry and antibodies

Tissue cryosections were rinsed in PBS to remove OCT and blocked at room temperature for 1 hour (5% BSA and 0.1% Triton X-100 in PBS). Subsequently, sections were incubated in the primary antibody for 1–2 hours. After washing 3 times (0.1% Triton X-100 in PBS), the samples were incubated in the secondary antibody in the blocking buffer for 1 hour at room temperature. Sections were mounted with DAPI to detect nuclei. Fluorescent images were captured using an upright Leica fluorescence microscope.

The following antibodies were used: chicken anti-GFP (1:1000, ab13970, Abcam, Cambridge, UK), mouse monoclonal anti-cTnT (1:1000, TI-1, DSHB, Iowa, USA), rat anti-CD31 (1:500, No.550274, BD PharMingen, Palo Alto, CA), rabbit monoclonal anti-PDGFRβ (1:200, ab32570, Abcam, Cambridge, UK), goat anti-Nkx2.5 (1:150, SC-8697, Santa Cruz Biotechnology, Santa Cruz, CA, USA), mouse monoclonal anti-α-SMA (1:500, A5228, Sigma-Aldrich, St Louis, MO, USA), rabbit anti-SM-MHC (1:250, BT-562, Biomedical Technologies Inc., Stoughton, MA, USA), rabbit polyclonal anti-Tbx18 (1:200, ab115262, Abcam, Cambridge, UK), mouse monoclonal anti-CD90 (1:200, ab225, Abcam, Cambridge, UK), mouse monoclonal anti-PDGFRα (1:200, ab96569, Abcam, Cambridge, UK) and WGA-Alexa488 (1:100, W6748, Invitrogen, USA).

The following secondary antibodies were used in this study: Alexa 594 donkey anti-rabbit IgG (A21207, Thermo Fisher, Grand Island, NY, USA), Alexa 488 goat anti-chicken IgG (A11039, Thermo Fisher), Alexa 594 donkey anti-goat IgG (A11058, Thermo Fisher), Alexa 594 donkey anti-rat IgG (A21209, Thermo Fisher), Alexa 594 goat anti-mouse IgG2a (A-21135, Thermo Fisher) and Dylight 488 rabbit anti-chicken IgY (ab96955, Abcam, Cambridge, UK). The secondary antibodies were diluted at 1:500 in blocking solution.

#### X-gal Staining

Hearts were harvested and fixed in 4% paraformaldehyde at 4°C for 25 minutes. They were transferred to sucrose solution and embedded in OCT compound as described above. Cryosections (12 μm) were prepared and stained for β-galactosidase activity with X-gal solution (50 mM K-ferricyanide, 50 mM K-ferrocyanide, 200 mM MgCl_2_, and 100 mg/ml X-gal in PBS) at 37°Cfor 12 hours. Sections were rinsed in PBS, counterstained and mounted as needed.

#### EdU pulse-chase analysis

EdU was dissolved in PBS and injected intraperitoneally into the mice (5 mg per 100 g body weight) after injury. The hearts were harvested on the 4th day after the last EdU injection. Immunodetection of the proliferative cells was performed using a Click-iT EdU Cell Proliferation Assay Kit (C35002, Thermo Fisher).

#### Cell apoptosis analysis

Tissue cryosections were rinsed in PBS to remove OCT and blocked at room temperature for 1 hour (5% BSA and 0.1% Triton X-100 in PBS). Apoptotic cells were labeled using the Click-iT Plus TUNEL Assay for *In Situ* Apoptosis Detection (C10617, Thermo Fisher).

### QUANTIFICATION AND STATISTICAL ANALYSIS

All data are presented as the mean ± standard error of the mean (SEM). Student’s unpaired t test was used for the comparisons between the sham and resected groups. A value of p < 0.05 was considered significant.

## Supplementary Material

1

## Figures and Tables

**Table:** KEY RESOURCES TABLE

REAGENT or RESOURCE	SOURCE	IDENTIFIER
Antibodies		
Chicken anti-GFP	Abcam	Cat# ab13970; RRID:AB_300798
Mouse monoclonal anti-cTnT	DSHB	Cat#TI-1; RRID:AB_2206413
Rat anti-CD31	BD PharMingen	Cat# 550274; RRID: AB_393571
Rabbit monoclonal anti-PDGFRβ	Abcam	Cat#ab32570; RRID:AB_777165
Mouse monoclonal anti-α-SMA	Sigma-Aldrich	Cat#A5228; RRID:AB_262054
Goat anti-Nkx2.5	Santa Cruz Biotechnology	Cat#SC-8697; RRID:AB_650280
Rabbit anti-SM-MHC	Biomedical Technologies Inc	Cat#BT-562; RRID:AB_10013421
Rabbit polyclonal anti-Tbx18	Abcam	Cat#ab115262
Mouse monoclonal anti-CD90	Abcam	Cat#ab225; RRID:AB_2203300
Mouse monoclonal anti-PDGFRα	Abcam	Cat#ab96569; RRID:AB_10687154
WGA-Alexa488	Invitrogen	Cat#W6748
Alexa594 donkey anti-rabbit IgG	Thermo Fisher	Cat#A21207; RRID:AB_141637
Alexa 488 goat anti-chicken IgG	Thermo Fisher	Cat#A11039; RRID:AB_142924
Alexa594 donkey anti-goat IgG	Thermo Fisher	Cat#A11058; RRID:AB_2534105
Alexa594 donkey anti-rat IgG	Thermo Fisher	Cat#A21209; RRID:AB_2535795
Alexa 594 goat anti-mouse IgG2a	Thermo Fisher	Cat#A21135; RRID:AB_1500827
Dylight 488 rabbit anti-chicken IgY	Abcam	Cat#Ab96955; RRID:AB_10679801
Chemicals, Peptides, and Recombinant Proteins		
Tamoxifen	Sigma-Aldrich	Cat#T5648
Bouin’s Solution	Sigma-Aldrich	Cat#HT10-1-32
Weigert’s Iron Hematoxylin Set	Sigma-Aldrich	Cat#HT10-79
5-Bromo-4-chloro-3-indolyl-α-D-galactopyranoside	Sigma-Aldrich	Cat#16555
Critical Commercial Assays		
Click-iT EdU Cell Proliferation Assay Kit	Thermo Fisher	Cat#C35002
Click-iT Plus TUNEL Assay for *In Situ* Apoptosis Detection	Thermo Fisher	Cat#C10617
Masson’s trichrome staining	Sigma-Aldrich	Cat#HT15-1KT
Experimental Models: Organisms/Strains		
Mouse: B6.129S4-Gt(ROSA)26Sor^tm1Sor^ /J	The Jackson Laboratory	JAX: 003474
Mouse: B6;129-Gt(ROSA)26Sor^tm9(CAG-GFP_*_)Nat^/J	The Jackson Laboratory	JAX: 026005
Mouse: B6;129-Gt(ROSA)26Sor^tm7(CAG-tdTomato_*_)Nat^/J	The Jackson Laboratory	JAX: 026006
Mouse: B6.129(Cg)-Gt(ROSA)26 Sor^tm4(ACTB-tdTomato,-EGFP)Luo^/J	The Jackson Laboratory	JAX: 007676
Mouse: *Tbx18^H2B-GFP^*	Cai et al., 2008	N/A
Mouse: *Tbx18*^MerCreMer/+^	This paper	N/A
Mouse: *SM-MHC*^CreERT2^	Wirth et al., 2008	N/A
Mouse: *Nfatc1*^Cre^	Wu et al., 2012	N/A
Software and Algorithms		
Photoshop CS software	Adobe	https://www.adobe.com/cn/products/cs6/photoshop.html

## Highlights

- Limited regeneration occurs in the neonatal mouse heart upon ventricular apex amputation
- Progenitor cell differentiation from epicardium is very minimal during heart repair
- New coronary vessels are generated through angiogenesis after injury

## References

[R1] Acharya A, Baek ST, Huang G, Eskiocak B, Goetsch S, Sung CY, Banfi S, Sauer MF, Olsen GS, Duffield JS, (2012). The bHLH transcription factor Tcf21 is required for lineage-specific EMT of cardiac fibroblast progenitors. Development 139, 2139–2149. 22573622 10.1242/dev.079970 PMC3357908

[R2] Andersen DC, Ganesalingam S, Jensen CH, and Sheikh SP (2014). Doneonatal mouse hearts regenerate following heart apex resection? Stem Cell Reports 2, 406–413. 24749066 10.1016/j.stemcr.2014.02.008 PMC3986579

[R3] Andersen DC, Jensen CH, Baun C, Hvidsten S, Zebrowski DC, Engel FB, and Sheikh SP (2016). Persistent scarring and dilated cardiomyopathy suggest incomplete regeneration of the apex resected neonatal mouse myocardium–A 180 days follow up study. J. Mol. Cell. Cardiol 90, 47–52. 26655949 10.1016/j.yjmcc.2015.11.031

[R4] Beltrami AP, Barlucchi L, Torella D, Baker M, Limana F, Chimenti S, Kasahara H, Rota M, Musso E, Urbanek K, (2003). Adult cardiac stem cells are multipotent and support myocardial regeneration. Cell 114, 763–776. 14505575 10.1016/s0092-8674(03)00687-1

[R5] Bergmann O, Bhardwaj RD, Bernard S, Zdunek S, Barnabé-Heider F, Walsh S, Zupicich J, Alkass K, Buchholz BA, Druid H, (2009). Evidence for cardiomyocyte renewal in humans. Science 324, 98–102. 19342590 10.1126/science.1164680 PMC2991140

[R6] Bryant DM, O’Meara CC, Ho NN, Gannon J, Cai L, and Lee RT (2015). A systematic analysis of neonatal mouse heart regeneration after apical resection. J. Mol. Cell. Cardiol 79, 315–318. 25533939 10.1016/j.yjmcc.2014.12.011 PMC4302033

[R7] Cai CL, Liang X, Shi Y, Chu PH, Pfaff SL, Chen J, and Evans S (2003). Isl1 identifies a cardiac progenitor population that proliferates prior to differentiation and contributes a majority of cells to the heart. Dev. Cell 5, 877–889. 14667410 10.1016/s1534-5807(03)00363-0 PMC5578462

[R8] Cai CL, Martin JC, Sun Y, Cui L, Wang L, Ouyang K, Yang L, Bu L, Liang X, Zhang X, (2008). A myocardial lineage derives from Tbx18 epicardial cells. Nature 454, 104–108. 18480752 10.1038/nature06969 PMC5540369

[R9] Cai X, Zhang W, Hu J, Zhang L, Sultana N, Wu B, Cai W, Zhou B, and Cai CL (2013). Tbx20 acts upstream of Wnt signaling to regulate endocardial cushion formation and valve remodeling during mouse cardiogenesis. Development 140, 3176–3187. 23824573 10.1242/dev.092502 PMC3931733

[R10] Chen HI, Sharma B, Akerberg BN, Numi HJ, Kivelä R, Saharinen P, Aghajanian H, McKay AS, Bogard PE, Chang AH, (2014). The sinus venosus contributes to coronary vasculature through VEGFC-stimulated angiogenesis. Development 141, 4500–4512. 25377552 10.1242/dev.113639 PMC4302936

[R11] Darehzereshki A, Rubin N, Gamba L, Kim J, Fraser J, Huang Y, Billings J, Mohammadzadeh R, Wood J, Warburton D, (2015). Differential regenerative capacity of neonatal mouse hearts after cryoinjury. Dev. Biol 399, 91–99. 25555840 10.1016/j.ydbio.2014.12.018 PMC4339535

[R12] Dawn B, Stein AB, Urbanek K, Rota M, Whang B, Rastaldo R, Torella D, Tang XL, Rezazadeh A, Kajstura J, (2005). Cardiac stem cells delivered intravascularly traverse the vessel barrier, regenerate infarcted myocardium, and improve cardiac function. Proc. Natl. Acad. Sci. USA 102, 3766–3771. 15734798 10.1073/pnas.0405957102 PMC553298

[R13] Haenig B, and Kispert A (2004). Analysis of TBX18 expression in chick embryos. Dev. Genes Evol 214, 407–411. 15257458 10.1007/s00427-004-0415-3

[R14] Han C, Nie Y, Lian H, Liu R, He F, Huang H, and Hu S (2015). Acute inflammation stimulates a regenerative response in the neonatal mouse heart. Cell Res 25, 1137–1151. 26358185 10.1038/cr.2015.110 PMC4650627

[R15] Haubner BJ, Adamowicz-Brice M, Khadayate S, Tiefenthaler V, Metzler B, Aitman T, and Penninger JM (2012). Complete cardiac regeneration in a mouse model of myocardial infarction. Aging (Albany N.Y.) 4, 966–977. 10.18632/aging.100526 PMC3615162 23425860

[R16] Huang GN, Thatcher JE, McAnally J, Kong Y, Qi X, Tan W, DiMaio JM, Amatruda JF, Gerard RD, Hill JA, (2012). C/EBP transcription factors mediate epicardial activation during heart development and injury. Science 338, 1599–1603. 23160954 10.1126/science.1229765 PMC3613149

[R17] Hudon-David F, Bouzeghrane F, Couture P, and Thibault G (2007). Thy-1 expression by cardiac fibroblasts: lack of association with myofibroblast contractile markers. J. Mol. Cell. Cardiol 42, 991–1000. 17395197 10.1016/j.yjmcc.2007.02.009

[R18] Ivey MJ, and Tallquist MD (2016). Defining the Cardiac Fibroblast. Circ. J 80, 2269–2276. 27746422 10.1253/circj.CJ-16-1003 PMC5588900

[R19] Jopling C, Sleep E, Raya M, Martí M, Raya A, and Izpisúa Belmonte JC (2010). Zebrafish heart regeneration occurs by cardiomyocyte dedifferentiation and proliferation. Nature 464, 606–609. 20336145 10.1038/nature08899 PMC2846535

[R20] Katz TC, Singh MK, Degenhardt K, Rivera-Feliciano J, Johnson RL, Epstein JA, and Tabin CJ (2012). Distinct compartments of the proepicardial organ give rise to coronary vascular endothelial cells. Dev. Cell 22, 639–650. 22421048 10.1016/j.devcel.2012.01.012 PMC3306604

[R21] Kikuchi K, Holdway JE, Werdich AA, Anderson RM, Fang Y, Egnaczyk GF, Evans T, Macrae CA, Stainier DY, and Poss KD (2010). Primary contribution to zebrafish heart regeneration by gata4(+) cardiomyocytes. Nature 464, 601–605. 20336144 10.1038/nature08804 PMC3040215

[R22] Kikuchi K, Gupta V, Wang J, Holdway JE, Wills AA, Fang Y, and Poss KD (2011). tcf21+ epicardial cells adopt non-myocardial fates during zebrafish heart development and regeneration. Development 138, 2895–2902. 21653610 10.1242/dev.067041 PMC3119303

[R23] Konfino T, Landa N, Ben-Mordechai T, and Leor J (2015). The type of injury dictates the mode of repair in neonatal and adult heart. J. Am. Heart Assoc 4, e001320. 25628406 10.1161/JAHA.114.001320 PMC4330059

[R24] Kotlikoff MI, Hesse M, and Fleischmann BK (2014). Comment on “Do neonatal mouse hearts regenerate following heart apex resection”? Stem Cell Reports 3, 2. 25068115 10.1016/j.stemcr.2014.06.010 PMC4110747

[R25] Kraus F, Haenig B, and Kispert A (2001). Cloning and expression analysis of the mouse T-box gene Tbx18. Mech. Dev 100, 83–86. 11118889 10.1016/s0925-4773(00)00494-9

[R26] Laflamme MA, and Murry CE (2011). Heart regeneration. Nature 473, 326–335. 21593865 10.1038/nature10147 PMC4091722

[R27] Leferovich JM, Bedelbaeva K, Samulewicz S, Zhang XM, Zwas D, Lankford EB, and Heber-Katz E (2001). Heart regeneration in adult MRL mice. Proc. Natl. Acad. Sci. USA 98, 9830–9835. 11493713 10.1073/pnas.181329398 PMC55538

[R28] Lepilina A, Coon AN, Kikuchi K, Holdway JE, Roberts RW, Burns CG, and Poss KD (2006). A dynamic epicardial injury response supports progenitor cell activity during zebrafish heart regeneration. Cell 127, 607–619. 17081981 10.1016/j.cell.2006.08.052

[R29] Li F, Wang X, Capasso JM, and Gerdes AM (1996). Rapid transition of cardiac myocytes from hyperplasia to hypertrophy during postnatal development. J. Mol. Cell. Cardiol 28, 1737–1746. 8877783 10.1006/jmcc.1996.0163

[R30] Limana F, Zacheo A, Mocini D, Mangoni A, Borsellino G, Diamantini A, De Mori R, Battistini L, Vigna E, Santini M, (2007). Identification of myocardial and vascular precursor cells in human and mouse epicardium. Circ. Res 101, 1255–1265. 17947800 10.1161/CIRCRESAHA.107.150755

[R31] Limana F, Bertolami C, Mangoni A, Di Carlo A, Avitabile D, Mocini D, Iannelli P, De Mori R, Marchetti C, Pozzoli O, (2010). Myocardial infarction induces embryonic reprogramming of epicardial c-kit(+) cells: role of the pericardial fluid. J. Mol. Cell. Cardiol 48, 609–618. 19968998 10.1016/j.yjmcc.2009.11.008

[R32] Madisen L, Zwingman TA, Sunkin SM, Oh SW, Zariwala HA, Gu H, Ng LL, Palmiter RD, Hawrylycz MJ, Jones AR, (2010). A robust and high-throughput Cre reporting and characterization system for the whole mouse brain. Nat. Neurosci 13, 133–140. 20023653 10.1038/nn.2467 PMC2840225

[R33] Mikawa T, and Fischman DA (1992). Retroviral analysis of cardiac morphogenesis: discontinuous formation of coronary vessels. Proc. Natl. Acad. Sci. USA 89, 9504–9508. 1409660 10.1073/pnas.89.20.9504 PMC50160

[R34] Mikawa T, and Gourdie RG (1996). Pericardial mesoderm generates a population of coronary smooth muscle cells migrating into the heart along with ingrowth of the epicardial organ. Dev. Biol 174, 221–232. 8631495 10.1006/dbio.1996.0068

[R35] Mollova M, Bersell K, Walsh S, Savla J, Das LT, Park SY, Silberstein LE, Dos Remedios CG, Graham D, Colan S, and Kühn B (2013). Cardiomyocyte proliferation contributes to heart growth in young humans. Proc. Natl. Acad. Sci. USA 110, 1446–1451. 23302686 10.1073/pnas.1214608110 PMC3557060

[R36] Muzumdar MD, Tasic B, Miyamichi K, Li L, and Luo L (2007). A global double-fluorescent Cre reporter mouse. Genesis 45, 593–605. 17868096 10.1002/dvg.20335

[R37] Naqvi N, Li M, Calvert JW, Tejada T, Lambert JP, Wu J, Kesteven SH, Holman SR, Matsuda T, Lovelock JD, (2014). A proliferative burst during preadolescence establishes the final cardiomyocyte number. Cell 157, 795–807. 24813607 10.1016/j.cell.2014.03.035 PMC4078902

[R38] Orlic D, Kajstura J, Chimenti S, Jakoniuk I, Anderson SM, Li B, Pickel J, McKay R, Nadal-Ginard B, Bodine DM, (2001). Bone marrow cells regenerate infarcted myocardium. Nature 410, 701–705. 11287958 10.1038/35070587

[R39] Polizzotti BD, Ganapathy B, Walsh S, Choudhury S, Ammanamanchi N, Bennett DG, dos Remedios CG, Haubner BJ, Penninger JM, and Kühn B (2015). Neuregulin stimulation of cardiomyocyte regeneration in mice and human myocardium reveals a therapeutic window. Sci. Transl. Med 7, 281ra45. 10.1126/scitranslmed.aaa5171 PMC5360874 25834111

[R40] Polizzotti BD, Ganapathy B, Haubner BJ, Penninger JM, and Kühn B (2016). A cryoinjury model in neonatal mice for cardiac translational and regeneration research. Nat. Protoc 11, 542–552. 26890681 10.1038/nprot.2016.031 PMC5464389

[R41] Porrello ER, and Olson EN (2014). A neonatal blueprint for cardiac regeneration. Stem Cell Res. (Amst.) 13 (3 Pt B), 556–570. 10.1016/j.scr.2014.06.003 PMC4316722 25108892

[R42] Porrello ER, Mahmoud AI, Simpson E, Hill JA, Richardson JA, Olson EN, and Sadek HA (2011). Transient regenerative potential of the neonatal mouse heart. Science 331, 1078–1080. 21350179 10.1126/science.1200708 PMC3099478

[R43] Poss KD, Wilson LG, and Keating MT (2002). Heart regeneration in zebrafish. Science 298, 2188–2190. 12481136 10.1126/science.1077857

[R44] Rodríguez CI, Buchholz F, Galloway J, Sequerra R, Kasper J, Ayala R, Stewart AF, and Dymecki SM (2000). High-efficiency deleter mice show that FLPe is an alternative to Cre-loxP. Nat. Genet 25, 139–140. 10835623 10.1038/75973

[R45] Sadek HA, Martin JF, Takeuchi JK, Leor J, Nie Y, Giacca M, and Lee RT (2014). Multi-investigator letter on reproducibility of neonatal heart regeneration following apical resection. Stem Cell Reports 3, 1. 25068114 10.1016/j.stemcr.2014.06.009 PMC4110774

[R46] Senyo SE, Steinhauser ML, Pizzimenti CL, Yang VK, Cai L, Wang M, Wu TD, Guerquin-Kern JL, Lechene CP, and Lee RT (2013). Mammalian heart renewal by pre-existing cardiomyocytes. Nature 493, 433–436. 23222518 10.1038/nature11682 PMC3548046

[R47] Smart N, Bollini S, Dubé KN, Vieira JM, Zhou B, Davidson S, Yellon D, Riegler J, Price AN, Lythgoe MF, (2011). De novo cardiomyocytes from within the activated adult heart after injury. Nature 474, 640–644. 21654746 10.1038/nature10188 PMC3696525

[R48] Smith CL, Baek ST, Sung CY, and Tallquist MD (2011). Epicardial-derived cell epithelial-to-mesenchymal transition and fate specification require PDGF receptor signaling. Circ. Res 108, e15–e26. 21512159 10.1161/CIRCRESAHA.110.235531 PMC3134964

[R49] Soonpaa MH, and Field LJ (1997). Assessment of cardiomyocyte DNA synthesis in normal and injured adult mouse hearts. Am. J. Physiol 272, H220–H226. 9038941 10.1152/ajpheart.1997.272.1.H220

[R50] Soonpaa MH, Kim KK, Pajak L, Franklin M, and Field LJ (1996). Cardiomyocyte DNA synthesis and binucleation during murine development. Am. J. Physiol 271, H2183–H2189. 8945939 10.1152/ajpheart.1996.271.5.H2183

[R51] Soriano P (1999). Generalized lacZ expression with the ROSA26 Cre reporter strain. Nat. Genet 21, 70–71. 9916792 10.1038/5007

[R52] Strungs EG, Ongstad EL, O’Quinn MP, Palatinus JA, Jourdan LJ, and Gourdie RG (2013). Cryoinjury models of the adult and neonatal mouse heart for studies of scarring and regeneration. Methods Mol. Biol 1037, 343–353. 24029946 10.1007/978-1-62703-505-7_20

[R53] Tian X, Pu WT, and Zhou B (2015). Cellular origin and developmental program of coronary angiogenesis. Circ. Res 116, 515–530. 25634974 10.1161/CIRCRESAHA.116.305097 PMC6914229

[R54] Travers JG, Kamal FA, Robbins J, Yutzey KE, and Blaxall BC (2016). Cardiac Fibrosis: The Fibroblast Awakens. Circ. Res 118, 1021–1040. 26987915 10.1161/CIRCRESAHA.115.306565 PMC4800485

[R55] Uygur A, and Lee RT (2016). Mechanisms of Cardiac Regeneration. Dev. Cell 36, 362–374. 26906733 10.1016/j.devcel.2016.01.018 PMC4768311

[R56] van Wijk B, Gunst QD, Moorman AF, and van den Hoff MJ (2012). Cardiac regeneration from activated epicardium. PLoS ONE 7, e44692. 23028582 10.1371/journal.pone.0044692 PMC3447884

[R57] Walsh S, Pontén A, Fleischmann BK, and Jovinge S (2010). Cardiomyocyte cell cycle control and growth estimation in vivo–an analysis based on cardiomyocyte nuclei. Cardiovasc. Res 86, 365–373. 20071355 10.1093/cvr/cvq005

[R58] Wirth A, Benyó Z, Lukasova M, Leutgeb B, Wettschureck N, Gorbey S, Orsy P, Horváth B, Maser-Gluth C, Greiner E, (2008). G12-G13-LARG-mediated signaling in vascular smooth muscle is required for salt-induced hypertension. Nat. Med 14, 64–68. 18084302 10.1038/nm1666

[R59] Wu B, Zhang Z, Lui W, Chen X, Wang Y, Chamberlain AA, Moreno-Rodriguez RA, Markwald RR, O’Rourke BP, Sharp DJ, (2012). Endocardial cells form the coronary arteries by angiogenesis through myocardial-endocardial VEGF signaling. Cell 151, 1083–1096. 23178125 10.1016/j.cell.2012.10.023 PMC3508471

[R60] Wu SP, Dong XR, Regan JN, Su C, and Majesky MW (2013). Tbx18 regulates development of the epicardium and coronary vessels. Dev. Biol 383, 307–320. 24016759 10.1016/j.ydbio.2013.08.019 PMC4172450

[R61] Zhang CH, and Kühn B (2014). Muscling up the heart: a preadolescent cardiomyocyte proliferation contributes to heart growth. Circ. Res 115, 690–692. 25258401 10.1161/CIRCRESAHA.114.304632 PMC4196263

[R62] Zhou B, Ma Q, Rajagopal S, Wu SM, Domian I, Rivera-Feliciano J, Jiang D, von Gise A, Ikeda S, Chien KR, and Pu WT (2008). Epicardial progenitors contribute to the cardiomyocyte lineage in the developing heart. Nature 454, 109–113. 18568026 10.1038/nature07060 PMC2574791

[R63] Zhou B, Honor LB, He H, Ma Q, Oh JH, Butterfield C, Lin RZ, Melero-Martin JM, Dolmatova E, Duffy HS, (2011). Adult mouse epicardium modulates myocardial injury by secreting paracrine factors. J. Clin. Invest 121, 1894–1904. 21505261 10.1172/JCI45529 PMC3083761

