# Supplementary material for: Limited Regeneration Potential with Minimal Epicardial Progenitor Conversions in the Neonatal Mouse Heart after Injury
Source: Cell Rep. Author manuscript; Available in PMC 2019 Nov 7. (PMC6837841; doi:10.1016/j.celrep.2019.06.003)
Supplement: 1 [file NIHMS1533535-supplement-1.pdf]

Cell Reports, Volume *28*

Supplemental Information

Limited Regeneration Potential with Minimal Epicardial Progenitor Conversions in the Neonatal Mouse Heart after Injury

Weibin Cai, Jing Tan, Jianyun Yan, Lu Zhang, Xiaoqiang Cai, Haiping Wang, Fang Liu, Maoqing Ye, and Chen-Leng Cai

Supplemental Information

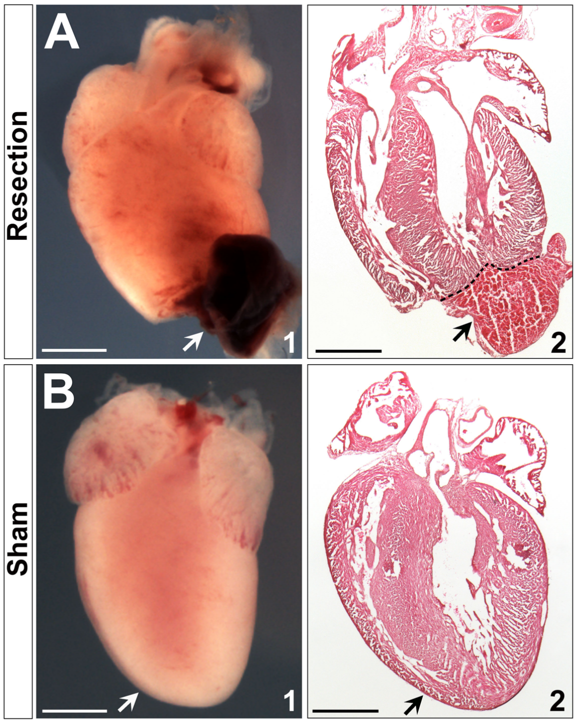

Figure S1. A typical ventricular apex amputation of the neonatal mouse heart, related to Figures 1 and 2.
(A) Amputation was performed on P1 heart by surgical resection in the ventricular apex (arrow). Following injury, a blood clot formed at the site to prevent exsanguination from the exposed ventricular chamber (arrow, 3h after surgery). **(B)** Ventricular apex in the sham group. A2 and B2 are transverse sections of the heart with H&E staining. The dashed line in A2 indicated the approximate amputation plane. Scale bar, 1mm.

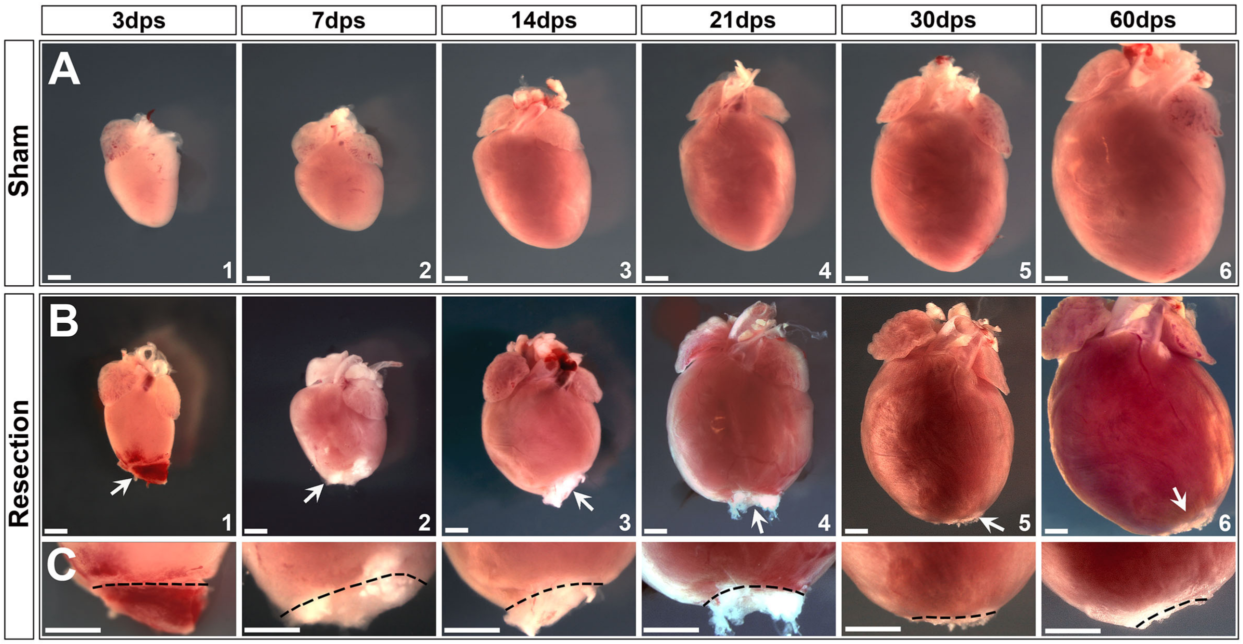

Figure S2. Whole-mount view of heart repair, related to Figures 1 and 2.
Sham **(A)** and apex-resected group **(B)** at 3, 7, 14, 21, 30 and 60 dps. Arrow denotes the injury site. **(C)** High magnification of the injured apex corresponding to B1-6. Scale bar, 1mm.

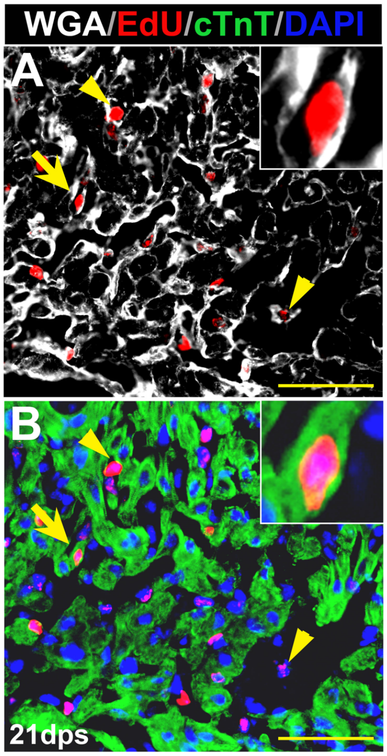

Figure S3. Proliferative cardiomyocyte after injury, related to Figure 3.
The neonatal mice were given ventricular apex resection at P1. Immunostaining was performed with antibodies to WGA, EdU and cTnT on hearts at 21dps. A and B show the same injury area with WGA/EdU (A) and EdU/cTnT/DAPI (B) staining, respectively. Arrows indicate proliferative mononuclear cardiomyocytes ($EdU^+/cTNT^+$), and arrowheads indicate proliferative non-cardiomyocytes ($EdU^+/cTNT^-$). The right-top corner in A and B are high magnification images of the proliferative mononuclear cardiomyocytes (arrow). Scale bar, 25 μm.

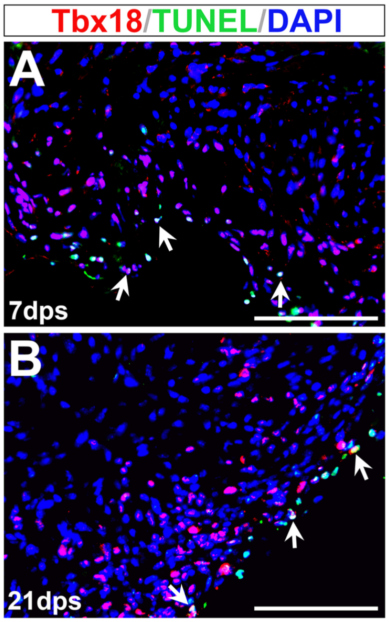

Figure S4. Tbx18^{+} cells exhibited apoptosis in injured area, related to Figure 4.
Ventricular apex resection was performed at P1 on neonatal mice. Immunostaining was performed on hearts at 7dps, 21dps and 60dps. Arrows in A/B indicate TUNEL and Tbx18 double-positive cells in the apex at 7dps (A), and 21dps (B). Scale bar, 100 µm.

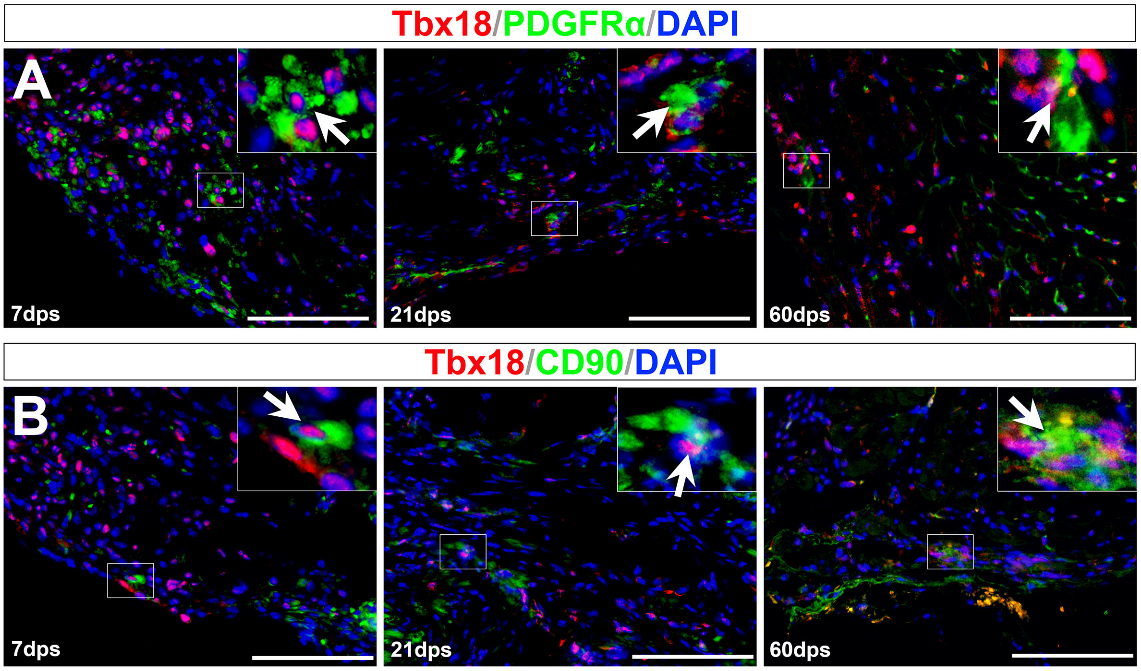

Figure S5. A subpopulation of Tbx18$^+$ cells are fibroblasts (PDGFRα$^+$ and CD90$^+$) in the injured apex, related to Figure 4.

Ventricular apex resection was performed at P1. Immunostaining with antibodies to Tbx18, PDGFRα and CD90 was performed at 7dps, 21dps and 60dps. The right-top corner are high magnification images of the areas outlined in each panel. Arrows in A indicate PDGFRα/Tbx18 double-positive cells in the apex at 7, 21 and 60 dps. Arrows in B indicate CD90/Tbx18 double-positive cells in the apex at 7, 21 and 60 dps. Scale bar, 100 µm.

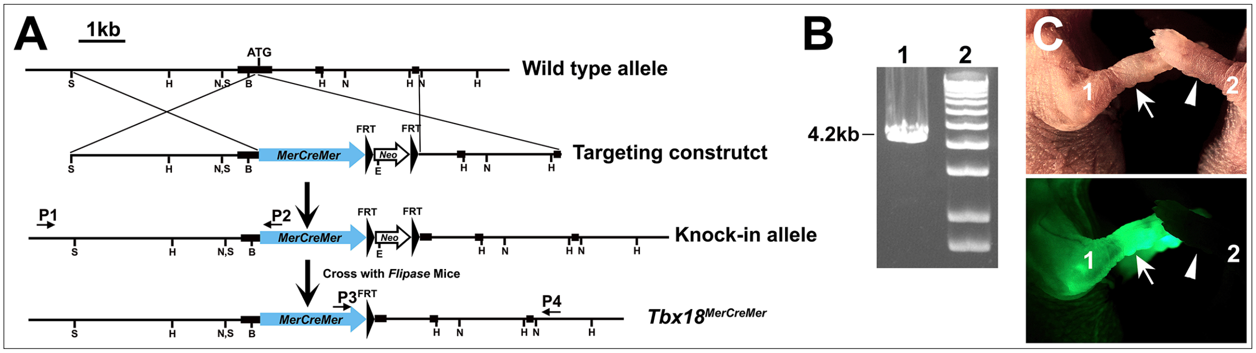

Figure S6. Generation of *Tbx18*^{MerCreMer} knock-in mice, related to Figures 5 and 6.
(A) A *MerCreMer-Neo* cassette was introduced into *Tbx18* exon 1 (6 bp upstream of the endogenous ATG, *Neo* cassette is flanked by *FRT* sites). *MerCreMer* is expressed under control of endogenous *Tbx18* promoter. **(B)** Long range PCR was performed with a primer external to the 5' targeting construct arm (P1) and a primer in *MerCerMer* cassette (P2). *Tbx18*$^{MerCreMer/+}$ mice were crossed to *Flippase* deleter mice (*FLPe*) to remove the *Neo* cassette. *Tbx18*$^{MerCreMer/+}$ is a heterozygous null allele (*Tbx18*$^{+/-}$) for *Tbx18* and do not show any developmental defects in a mixed genetic background (Black Swiss). H, *Hind*III; S, *Sma*I; E, *Eco*RI. **(C)** *Tbx18*$^{MerCreMer/+}$;*R26R*$^{GFP/+}$ littermates with (C1) or without (C2) tamoxifen induction at P0. GFP was detected 1 day after induction (arrow in C1), and was not detected on littermate without tamoxifen (arrowhead in C2). Upper panel is bright-field and lower panel is under fluorescence illumination.
